# Differences in hand hygiene knowledge and perceptions among nursing students and registered nurses during and after the COVID-19 pandemic: A comparative cross-sectional survey

**DOI:** 10.1016/j.ijnsa.2026.100639

**Published:** 2026-07-20

**Authors:** Per-Ola Blomgren, Lisa Hultin, Johan Westerbergh, Katarina Hjelm

**Affiliations:** a Department of Public Health and Caring Sciences, Uppsala University, Uppsala, Sweden; b Uppsala Clinical Research Center, Uppsala University, Uppsala, Sweden

**Keywords:** Cross-sectional, Hand hygiene, Infection prevention and control, Nursing education;Nursing students, Registered nurses

## Abstract

**Background:**

Hand hygiene is a cornerstone of infection prevention and control and plays a critical role in preventing healthcare-associated infections. During the COVID-19 pandemic, hand hygiene awareness and adherence increased across healthcare systems, but evidence on whether these improvements were sustained as pandemic pressures subsided remains limited. Nursing students and registered nurses are key groups for understanding long-term behavioural changes, as their knowledge, perceptions, and workplace experiences influence patient safety practices.

**Objective:**

To compare nursing students’ and registered nurses’ knowledge and perceptions of hand hygiene at two time points: during the peak of the COVID-19 pandemic in 2020 and in the post-pandemic period in 2023.

**Design:**

Comparative cross-sectional survey study conducted at two time points (2020 and 2023).

**Setting:**

A Swedish university and its affiliated university hospital.

**Participants:**

A total of 376 individuals: first-semester nursing students, final-semester nursing students, and registered nurses surveyed in 2020 (n = 201) and 2023 (n = 175).

**Methods:**

This study employed a two-time point, independent-sample design. Data were collected online using two validated World Health Organization instruments: the Hand Hygiene Knowledge Questionnaire for Health Care Workers and the Perception Survey for Health Care Workers. Descriptive statistics were computed for all variables. Comparisons between 2020 and 2023 were performed using the Mann–Whitney U test for ordinal variables and Fisher’s exact test for categorical variables.

**Results:**

Total knowledge scores did not differ significantly between time points. However, first-semester students had lower knowledge of transmission routes and preventive actions in 2023. Perceptions of hand hygiene differed between time points. The mean score for perceived importance of hand hygiene as a patient safety measure was significantly lower in 2023 than in 2020 for first-semester students (3.77 vs 3.30, p < 0.001), final-semester students (3.61 vs 2.89, p < 0.001), and registered nurses (3.39 vs 3.19, p = 0.041). Perceived patient expectations about hand hygiene were also lower among nursing students, and registered nurses rated perceived hand hygiene adherence among healthcare workers lower in 2023. By contrast, first-semester students reported higher ratings of educational support and tolerance of alcohol-based hand rubs in 2023.

**Conclusions:**

The findings indicate group-level differences in hand hygiene perceptions between the pandemic-period and post-pandemic samples, while total knowledge scores did not differ significantly within any participant group. Given the independent-sample design, these findings should not be interpreted as individual-level changes over time. Lower perceived importance of hand hygiene suggests a need for continued educational reinforcement and organisational support to maintain hand hygiene practices.

**What is already known**

• Hand hygiene adherence increased during the COVID-19 pandemic.
• Hand hygiene knowledge and perceptions vary by education and clinical experience.
• Evidence on hand hygiene knowledge and perceptions among nursing students and registered nurses in the post-pandemic period remains limited.

Alt-text: Unlabelled box dummy alt text

**What this paper adds**

• Post-pandemic hand hygiene perceptions were less favourable, particularly perceived importance, patient expectations, and perceived adherence.
• Total knowledge scores were similar, but education should target selected knowledge gaps and reinforce perceived hand hygiene importance, patient expectations, and perceived adherence.

Alt-text: Unlabelled box dummy alt text

## 1 Background

Hand hygiene is a cornerstone of infection prevention and control, playing a vital role in reducing healthcare-associated infections and protecting patients, healthcare workers, and visitors. Nurses, as frontline caregivers, play a central role in promoting and maintaining hand hygiene practices, which aligns with the World Health Organization (WHO) guidance on healthcare workers responsibilities for infection prevention and control (World Health Organization, 2024; WHO, 2009) and the International Council of Nurses’ “Code of Ethics for Nurses”, which highlights nurses’ professional duty to safeguard patients, model safe care, and collaborate with colleagues to uphold infection prevention and control standards (ICN - International Council of Nurses [Internet], 2023). However, achieving consistent adherence requires more than theoretical knowledge; it demands behavioural commitment, reinforced by leadership, education, and a strong culture of patient safety (World Health Organization, 2024).

The emergence of coronavirus disease 2019 (COVID-19), beginning in December 2019, presented unprecedented challenges to healthcare systems worldwide, including increased patient loads, heightened infection risks among healthcare workers (Filip et al., 2022), and shortages of personal protective equipment (Shortage of personal protective equipment endangering health workers worldwide [Internet], 2024). The pandemic underscored the importance of hand hygiene, leading to improved adherence during its peak (Filip et al., 2022; Roshan et al., 2020; Makhni et al., 2021). However, as healthcare operations normalised following the World Health Organization’s declaration on 5 May 2023 that COVID-19 was no longer a public health emergency of international concern (hereinafter referred to as the post-pandemic period) (Statement on the fifteenth meeting of the IHR, 2005), maintaining these improvements has been considered challenging (Makhni et al., 2021; Guerrero-Soler et al., 2024; Organization and Fund, 2021).

In 2020, during the peak of the pandemic in Sweden (Socialstyrelsen [Internet], 2024), two studies examining hand hygiene among nursing students and registered nurses were conducted (Blomgren et al., 2024, 2025). The first study focused on hand hygiene knowledge (Blomgren et al., 2024) using the WHO Hand Hygiene Knowledge Questionnaire (WHO, 2009), while the second investigated attitudes and perceptions of hand hygiene (Blomgren et al., 2025) using the WHO Perception Survey for Health-Care Workers (WHO, 2009). These studies identified variations in both knowledge and perceptions between first- and final-semester nursing students and registered nurses. More specifically, final-semester students and registered nurses had higher hand hygiene knowledge scores than first-semester students in 2020, indicating that knowledge differed by educational stage and clinical experience (Blomgren et al., 2024). The perception study showed that first-semester students rated hand hygiene more positively than registered nurses on several items, including the importance of hand hygiene for patient safety and support from the educational or institutional context, whereas registered nurses reported higher perceived adherence to recommended hand hygiene (Blomgren et al., 2025). These 2020 results provide the pandemic-period comparison data for the present study.

Nursing students are a particularly relevant group, as their early professional experiences strongly influence the development of long-term infection prevention and control behaviours (Zimmerman et al., 2020). The results showed that final-semester students and registered nurses scored higher on knowledge than first-semester students (Blomgren et al., 2024), while attitudes and perceptions of hand hygiene were influenced by perceived institutional support and peer expectations (Blomgren et al., 2025).

Previous research among nursing students suggests that positive attitudes towards hand hygiene are associated with higher adherence to recommended hand hygiene protocols (Zimmerman et al., 2020), while factors such as workplace norms, peer influence, and organisational culture further shape hand hygiene behaviour (Barrett and Randle, 2008; Labrague et al., 2018).

Sustaining hand hygiene practices requires a combination of resource availability, education, monitoring, and behavioural reinforcement (World Health Organization, 2024). Consistent access to hand rubs, clean water, and clear infection prevention and control guidelines is fundamental. Training programmes, workplace reminders, and structured feedback mechanisms further support implementation. Beyond these practical measures, fostering a strong safety culture is crucial (World Health Organization, 2024). Recent practice recommendations also stress systematic monitoring with feedback, accountability mechanisms, and the protection of skin health through the use of effective, well-tolerated products as essential components for maintaining long-term adherence to hand hygiene (Glowicz et al., 2022). Previous reviews highlight the influence of social norms, self-efficacy, and peer reinforcement, underscoring the importance of leadership and visible role modelling in sustaining adherence (Luangasanatip et al., 2015; Huis et al., 2012). Regular feedback plays a key role in maintaining hand hygiene adherence; a previous study found that healthcare workers not only accepted electronic reminders but also expressed a clear desire for more frequent and structured feedback on their performance (Blomgren et al., 2021).

The Theory of Planned Behaviour provides a useful framework for understanding hand hygiene adherence (Ajzen, 1991). According to the Theory of Planned Behaviour, behaviour is determined by three core components: attitudes towards the behaviour, subjective norms (social expectations), and perceived behavioural control (the perceived ease or difficulty of performing the behaviour). In the context of hand hygiene, individual beliefs may be based on knowledge and refined through experiences, thereby shaping attitudes towards hand hygiene (Hadziabdic et al., 2020). The institutional context (e.g. university education and hospital training) provides formal learning structures that shape how hand hygiene is perceived and understood. The social context (e.g. colleagues and peers) contributes through observation, interaction, and professional socialisation. Thus, knowledge acquired through both formal education and clinical experience provides the foundation for understanding when and why hand hygiene is required, while personal experiences further reinforce or reshape beliefs that guide attitudes and hand hygiene behaviour. This dynamic feedback loop supports ongoing learning and behavioural adaptation (Hadziabdic et al., 2020).

In summary, the ongoing challenge of combating healthcare-associated infections highlights the critical importance of promoting hand hygiene adherence in healthcare settings. The COVID-19 pandemic placed an unprecedented spotlight on infection prevention and control measures, emphasising hand hygiene as vital for the safety of patients, healthcare workers, and visitors. Studies conducted during the COVID-19 pandemic reported changes in hand hygiene practices among healthcare workers, including increased adherence during periods of high transmission (Makhni et al., 2021; Guerrero-Soler et al., 2024) and heightened organisational focus on infection prevention (Filip et al., 2022; Organization and Fund, 2021).

Against this background, two studies conducted in Sweden during the peak of the pandemic, using validated WHO instruments, identified significant variations in knowledge and attitudes towards hand hygiene among nursing students and registered nurses (Blomgren et al., 2024, 2025). However, it remains unclear whether hand hygiene knowledge and perceptions among nursing students and registered nurses differ between the pandemic and post-pandemic periods when assessed using comparable methods. To date, no studies have examined how knowledge and perceptions of hand hygiene have evolved among these groups following the acute phase of the pandemic using a comparable methodology across time points. Therefore, this study aimed to compare nursing students’ and registered nurses’ knowledge and perceptions of hand hygiene during the peak of the COVID-19 pandemic in 2020 and the post-pandemic period in 2023. It was hypothesised that knowledge and perceptions of hand hygiene would differ in the post-pandemic period.

## 2 Methods

### 2.1 Study design

A descriptive cross-sectional total survey design was used to compare hand hygiene knowledge and perceptions at two time points in 2020 and 2023.

### 2.2 Study setting and sampling

The study was conducted at a Swedish university offering a three-year nursing programme (six semesters) and its affiliated university hospital. Three participant groups were targeted: first-semester nursing students, who had received introductory infection prevention and control education and completed an initial clinical placement; final-semester nursing students, who had completed advanced infection prevention and control coursework and accumulated broader clinical experience; and registered nurses working in geriatric, medical, or surgical wards at the university hospital. The infection prevention and control training content and focus across these educational stages and the registered nurse’s workplace are summarised in Supplementary Table 1.

### 2.3 Instruments

Data were collected using two WHO instruments: The Hand Hygiene Knowledge Questionnaire for Health-Care Workers (WHO, 2009) assessed knowledge of hand hygiene indications, transmission, and preventive strategies using 25 questions: 12 yes/no, four true/false, and nine multiple-choice items. Each correct response was scored as 1 and each incorrect or missing response as 0, giving a total possible score of 0–25, with higher scores indicating greater knowledge. The Perception Survey for Health-Care Workers (WHO, 2009) evaluated perceptions of hand hygiene, patient safety, perceived effectiveness of improvement strategies, and self-reported hand hygiene behaviour. This survey comprised 24 questions based on parts 1 and 2 of the original WHO tool, primarily using Likert-scale and numeric response formats. Because the perception survey includes items with different response formats and scale ranges, including four-point rating scales, seven-point rating scales, and percentage estimates from 0 to 100, no total perception score was calculated. Instead, each item was analysed separately to identify which specific aspects of hand hygiene perceptions differed between 2020 and 2023.

The validity of the instruments was supported by their development by the WHO for use in hand hygiene evaluation and improvement (WHO, 2009). The Swedish versions had previously been translated and adapted (Blomgren et al., 2025) according to WHO recommendations, including forward–backward translation, expert panel review, and pilot testing (World Health Organization). The pilot testing with nursing students and registered nurses (n = 20) resulted in minor contextual modifications to suit student and registered nurse roles (Blomgren et al., 2025).

### 2.4 Data collection

The survey followed a two-time-point, independent-sample design and was administered online to nursing students in their first- and final-semester and registered nurses. Data were collected between December 2020 and January 2021, during the peak of the COVID-19 pandemic (Socialstyrelsen [Internet], 2024), and in May 2023, during the post-pandemic period (Statement on the fifteenth meeting of the IHR, 2005).

The same web-based survey platform and recruitment approach were used for both study periods. An information letter was emailed to all eligible participants one week prior to survey distribution. During each one-month collection period, two reminders were sent via institutional email channels. Nursing students were additionally reminded during lectures by a member of the research team, and registered nurses were contacted through heads of the departments and administrative staff.

### 2.5 Data analysis

Descriptive statistics were used to summarise the data, including frequencies, percentages, means with standard deviations, and medians with interquartile ranges, in line with standard methodological guidance (Petrie and Sabin, 2020). Analyses were conducted separately for first-semester nursing students, final-semester nursing students, and registered nurses. Differences between the 2020 and 2023 datasets were assessed using the Mann–Whitney U test for ordinal variables and Fisher’s exact test for categorical variables (Petrie and Sabin, 2020). A p-value of < 0.05 was considered statistically significant. All analyses were performed using the Statistical Package for the Social Sciences, version 28 (SPSS Inc., Chicago, IL, USA).

### 2.6 Ethical considerations

This study adhered to the principles of the Declaration of Helsinki (World Medical Association, 2013) and applicable Swedish legislation (Riksdagsförvaltningen, 2021). Ethical review was not required, as the study did not involve the processing of sensitive personal data and posed no physical or psychological risk to participants. Necessary permissions were obtained from the head of the nursing programme at the university and the chief nurse and heads of the participating hospital departments. The web-based questionnaire provided clear information about the study’s purpose, the voluntary nature of participation, and data confidentiality. Participants were informed that submission of the completed questionnaire implied consent to participate. This information was presented at the beginning of the questionnaire to ensure that consent was fully informed. All responses were anonymised, treated with strict confidentiality to protect participants’ privacy, and stored according to the university regulations.

## 3 Results

### 3.1 Characteristics of the participants

In total, 950 individuals were invited to participate (2020 and 2023 combined), with 376 responding, yielding an overall response rate of 39.6%. In 2020, the response rates were 56% among first-semester nursing students (71/128), 57% among final-semester nursing students (46/81), and 30% among registered nurses (84/278). In 2023, the corresponding response rates were 35% among first-semester nursing students (49/140), 46% among final-semester nursing students (46/101), and 36% among registered nurses (80/222).

Across both study years (2020 and 2023), the majority of participants were female, ranging from 78.9% to 90% across groups. Median age was similar between the two time points among first-semester nursing students, final-semester nursing students, and registered nurses. Participant characteristics are presented in Table 1.

**Table 1:** Demographic characteristics of the study population. First-semester nursing students, final-semester nursing students, and registered nurses across the 2020 and 2023 study periods.

	First-semester nursing students		Final-semester nursing students		Registered nurses	
Variable	2020 (*n* = 71)	2023 (*n* = 49)	2020 (*n* = 46)	2023 (*n* = 46)	2020 (*n* = 84)	2023 (*n* = 80)
Age^1^	22 (20.00–25.25)	22 (21.00–27.50)	26 (23.00–29.25)	24 (22.00–26.00)	34 (28.00–46.75)	36 (30.00–47.00)
Female n (%)	56 (78.9)	39 (79.6)	40 (87)	37 (80.4)	73 (86.9)	72 (90)
Male n (%)	15 (21.1)	10 (20.4)	6 (Blomgren et al., 2025)	9 (19.6)	11 (13.1)	8 (Organization and Fund, 2021)
Experience of work as an assistant nurse, n (%)	29 (Polit and Beck, 2021)	26 (55.3)	34 (73.9)	37 (80.4)		
Working experience as a nurse^1^					9 (2.50–16.00)	10 (4.00–15.00)
Formal training in hand hygiene in the last three years, n (%)	48 (67.6)	42 (85.7)	39 (84.7)	38 (84.4)	51 (60.7)	39 (49.4)

1 Years, median (IQR)

Prior work experience as an assistant nurse was more commonly reported in 2023 than in 2020 among first-semester nursing students (55.3% vs 42.0%) and final-semester nursing students (80.4% vs 73.9%).

Regarding formal hand hygiene training, the proportion reporting training within the past three years was significantly higher among first-semester nursing students in 2023 than in 2020 (86.0% vs. 68.0%, p = 0.032). No significant differences were observed for final-semester nursing students (∼85% in both years, p = 1.000) or registered nurses (61% in 2020 vs 49% in 2023, p = 0.115).

### 3.2 Comparison of knowledge regarding hand hygiene

No significant differences in mean total knowledge scores were found between the 2020 and 2023 samples within first-semester nursing students, final-semester nursing students or registered nurses (Table 2).

**Table 2:** Total scores from the 25-question knowledge survey among first-semester nursing students, final-semester nursing students and registered nurses in 2020 and 2023.

	First-semester nursing students			Final-semester nursing students			Registered nurses		
Year	2020 (*n* = 71)	2023 (*n* = 49)	p value (U)	2020 (*n* = 46)	2023 (*n* = 46)	p value (U)	2020 (*n* = 84)	2023 (*n* = 80)	p value (U)
Total score	17.07 ± 2.02	16.76 ± 2.45	0.726 (1685)	18.76 ± 1.71	18.15 ± 1.90	0.619 (884.5)	18.38 ± 2.18	18.30 ± 1.89	0.269 (3321)

Note. Values are presented as mean ± standard deviation (SD). Total scores range from 0 to 25, with higher scores indicating greater hand hygiene knowledge. Comparisons between the 2020 and 2023 samples were performed separately within each participant group using the Mann–Whitney U test. For each comparison, the p value is reported together with the corresponding Mann–Whitney U value in parentheses.

Table 3 presents item-level knowledge results and identifies specific differences between the 2020 and 2023 samples. At item level, statistically significant differences were observed among first-semester nursing students (Supplementary Table 2). In this group, correct responses were lower in 2023 than in 2020 for items concerning the routine use of alcohol-based hand disinfectants (80.0% vs 94.0%, p = 0.022), the main route of cross-transmission (78.0% vs 93.0%, p = 0.026), avoiding jewellery (90.0% vs 100.0%, p = 0.010) and recognising artificial fingernails as a risk factor for germ colonisation (92.0% vs 100.0%, p = 0.028). No statistically significant item-level differences were observed among final-semester nursing students or registered nurses.

**Table 3:** Item-level results from the Hand Hygiene Knowledge Questionnaire for Health-Care Workers among first-semester nursing students, final-semester nursing students, and registered nurses in 2020 and 2023.

	First-semester nursing students			Final-semester nursing students			Registered nurses		
Item	2020 (*n* = 71)	2023 (*n* = 49)	p-value	2020 (*n* = 46)	2023 (*n* = 46)	p-value	2020 (*n* = 84)	2023 (*n* = 80)	p-value
Formal training in hand hygiene within the last three years	68 [0]	86 [0]	0.032	85 [0]	85 [1]	1.000	61 [1]	49 [1]	0.115
Routinely used an alcohol-based hand disinfectant in healthcare settings	94 [3]	80 [0]	0.022	100 [0]	93 [0]	0.242	100 [0]	100 [0]	N/A
Main route of cross-transmission of potentially harmful germs between patients in a healthcare facility	93 [0]	78 [0]	0.026	91 [0]	91 [0]	1.000	94 [0]	92 [0]	0.762
Most frequent source of germs responsible for healthcare-associated infections	49 [0]	49 [0]	1.000	46 [0]	59 [0]	0.297	48 [1]	58 [1]	0.212
The following hand hygiene actions prevent the transmission of germs to patients, answered with yes or no alternatives									
Before touching a patient	99 [0]	98 [0]	1.000	98 [0]	98 [0]	1.000	100 [0]	98 [0]	0.236
Immediately after a risk of body fluid exposure	10 [3]	2 [0]	0.136	15 [0]	4 [1]	0.158	10 [3]	8 [0]	0.781
After exposure to the immediate surroundings of a patient	9 [3]	4 [0]	0.465	11 [0]	4 [0]	0.434	7 [2]	5 [0]	0.746
Immediately before a clean/aseptic procedure	89 [1]	86 [0]	0.780	100 [0]	98 [0]	1.000	100 [2]	98 [0]	0.494
The following hand hygiene actions prevent the transmission of germs to healthcare workers, answered with yes or no alternatives									
After touching a patient	91 [2]	94 [0]	0.734	100 [1]	96 [0]	0.495	99 [0]	98 [0]	0.614
Immediately after a risk of body fluid exposure	99 [2]	96 [0]	0.569	96 [0]	100 [0]	0.495	100 [1]	96 [0]	0.116
Immediately before a clean/aseptic procedure	13 [2]	12 [0]	1.000	32 [2]	17 [0]	0.143	17 [2]	11 [1]	0.370
After exposure to the immediate surroundings of a patient	100 [1]	94 [0]	0.067	100 [0]	98 [0]	1.000	100 [0]	100 [0]	N/A
True statements about alcohol-based disinfectant and handwashing with soap and water are answered with true or false alternatives									
Hand rubbing is more rapid for hand cleansing than handwashing	53 [3]	55 [0]	0.853	62 [1]	65 [0]	0.829	46 [1]	46 [0]	1.000
Hand rubbing causes skin dryness more than handwashing	69 [4]	77 [0]	0.400	89 [3]	73 [1]	0.052	90 [1]	90 [0]	1.000
Hand rubbing is more effective against germs than handwashing	56 [3]	59 [0]	0.850	47 [1]	47 [1]	1.000	53 [1]	50 [1]	0.875
Hand washing and hand disinfectant are recommended to be performed in sequence	21 [0]	22 [0]	1.000	7 [1]	11 [0]	0.714	10 [1]	9 [0]	1.000
Minimal time needed for alcohol-based handrub to kill most germs on your hands	68 [0]	51 [0]	0.087	76 [0]	70 [0]	0.640	79 [0]	85 [2]	0.419
Type of hand hygiene method required, answered with the alternatives: hand disinfectant, handwashing, or none									
Before palpation of the abdomen	71 [0]	72 [1]	0.838	96 [0]	87 [0]	0.267	95 [0]	86 [0]	0.059
Before giving an injection	94 [1]	96 [1]	1.000	87 [0]	98 [0]	0.111	99 [1]	96 [0]	0.361
After emptying a bedpan	67 [1]	65 [1]	0.844	84 [1]	74 [0]	0.303	70 [1]	70 [0]	1.000
After removing examination gloves	89 [1]	83 [1]	0.426	98 [0]	96 [0]	1.000	98 [1]	95 [0]	0.437
After making a patient’s bed	65 [2]	79 [1]	0.147	85 [1]	74 [0]	0.303	78 [1]	79 [1]	1.000
After visible exposure to blood	73 [0]	73 [1]	1.000	94 [0]	80 [1]	0.069	82 [0]	84 [0]	0.838
The following should be avoided to prevent the colonisation of hands with harmful germs, answered with yes or no alternatives									
Wearing jewelry	100 [1]	90 [0]	0.010	100 [0]	100 [0]	N/A	100 [0]	100 [0]	N/A
Damaged skin	94 [1]	96 [0]	1.000	100 [1]	98 [0]	1.000	96 [0]	100 [0]	0.246
Artificial fingernails	100 [2]	92 [0]	0.028	100 [1]	100 [0]	N/A	100 [2]	100 [0]	N/A
Regular use of hand cream	68 [3]	61 [0]	0.557	84 [1]	84 [1]	1.000	81 [0]	80 [0]	1.000

Note. Values are presented as the percentage of correct responses. Missing responses are shown as n in square brackets. Comparisons between the 2020 and 2023 samples were performed separately within each participant group using Fisher’s exact test. For each comparison, the p value is reported.

### 3.3 Comparison of perceptions towards hand hygiene

Table 4 presents item-level results from the WHO Perception Survey. No total perception score was calculated; therefore, all comparisons refer to individual survey items between the 2020 and 2023 samples within each participant group.

**Table 4:** Item-level responses from the WHO Perception Survey for Health-Care Workers among first-semester nursing students, final-semester nursing students, and registered nurses in 2020 and 2023.

	First-semester nursing students			Final-semester nursing students			Registered nurses		
Item	2020 (*n* = 71)	2023 (*n* = 49)	p value (U)	2020 (*n* = 46)	2023 (*n* = 46)	p value (U)	2020 (*n* = 84)	2023 (*n* = 80)	p value (U)
Impact of a healthcare-associated infection on a patient's clinical outcome^a^	2.94 ± 0.54	3.09 ± 0.58	0.176 (1425.5)	3.20 ± 0.58	3.27 ± 0.50	0.606 (981.0)	3.10 ± 0.64	3.14 ± 0.55	0.860 (3156.5)
Effectiveness of hand hygiene in preventing healthcare-associated infection^a^	3.86 ± 0.35	3.94 ± 0.25	0.173 (1545.0)	3.91 ± 0.29	3.82 ± 0.39	0.203 (941.0)	3.71 ± 0.55	3.75 ± 0.54	0.555 (3189.5)
Importance of hand hygiene on a ward among all patient safety issues^b^	3.77 ± 0.487	3.30 ± 0.832	<0.001 (1128.0)	3.61 ± 0.537	2.89 ± 0.722	<0.001 (479.5)	3.39 ± 0.807	3.19 ± 0.765	0.041 (2791.0)
Leaders and senior managers at your institution support and advocate for hand hygiene^c^	6.01 ± 1.15	5.90 ± 1.08	0.445 (1503.0)	5.54 ± 1.28	5.45 ± 1.44	0.957 (1005.5)	5.51 ± 1.48	5.54 ± 1.30	0.880 (3315.5)
The healthcare facility ensures that alcohol-based handrub is available^c^	6.66 ± 0.74	6.35 ± 1.21	0.207 (1498.0)	6.52 ± 0.91	6.42 ± 0.81	0.215 (902.0)	6.47 ± 0.93	6.61 ± 0.83	0.300 (3070.0)
Hand hygiene posters are displayed at point of care as reminders^c^	5.46 ± 1.48	5.27 ± 1.44	0.420 (1536.5)	4.74 ± 1.71	5.20 ± 1.38	0.207 (879.0)	4.60 ± 1.69	4.99 ± 1.76	0.126 (2903.5)
Each healthcare worker receives education on hand hygiene^c^	6.66 ± 0.61	6.54 ± 0.71	0.408 (1559.0)	6.43 ± 0.96	6.20 ± 1.18	0.274 (915.0)	6.18 ± 1.17	6.30 ± 0.97	0.704 (3215.0)
Visible. simple and clear instructions for hand hygiene are available for every healthcare worker^c^	6.17 ± 1.10	6.33 ± 0.93	0.556 (1560.0)	5.87 ± 1.38	5.93 ± 1.10	0.841 (1011.0)	5.54 ± 1.55	5.98 ± 1.19	0.104 (2744.5)
Healthcare workers regularly receive feedback on their hand hygiene performance^c^	6.26 ± 0.92	6.02 ± 1.31	0.568 (1506.0)	5.89 ± 1.65	5.67 ± 1.49	0.212 (847.0)	5.80 ± 1.49	6.03 ± 1.21	0.472 (3076.5)
You always perform hand hygiene as recommended (being a good example)^c^	6.27 ± 1.01	6.19 ± 0.96	0.490 (1566.0)	6.15 ± 1.15	5.96 ± 1.00	0.203 (884.5)	6.10 ± 1.04	6.06 ± 1.06	0.839 (3262.5)
Patients are invited to remind healthcare workers to perform hand hygiene^c^	4.06 ± 2.03	4.74 ± 1.92	0.073 (1287.0)	4.55 ± 2.23	4.20 ± 1.88	0.315 (869.0)	4.61 ± 1.95	4.57 ± 1.98	0.943 (3338.5)
Organisational importance on optimal hand hygiene^d^	6.13 ± 1.02	6.52 ± 0.68	0.030 (1320.0)	5.69 ± 1.28	5.73 ± 1.13	0.945 (982.0)	5.33 ± 1.56	5.16 ± 1.63	0.547 (3027.5)
Importance of peers’ and educators' commitment to optimal hand hygiene^d^	6.07 ± 1.06	6.29 ± 0.87	0.324 (1491.5)	5.15 ± 1.40	5.61 ± 1.32	0.061 (787.0)	5.17 ± 1.20	5.03 ± 1.55	0.793 (3164.5)
Importance that patients place on optimal hand hygiene d	5.55 ± 1.44	4.96 ± 1.62	0.050 (1252.0)	5.04 ± 1.87	3.48 ± 1.62	<0.001 (550.5)	4.19 ± 1.81	4.29 ± 1.88	0.724 (3254.0)
Effort required to perform good hand hygiene^e^	3.23 ± 1.74	3.29 ± 2.23	0.713 (1614.0)	2.65 ± 1.52	2.61 ± 1.53	0.825 (985.5)	2.34 ± 1.63	2.39 ± 1.89	0.559 (3115.0)
Alcohol-based handrubs tolerated by hands^f^	5.35 ± 1.61	5.96 ± 1.20	0.042 (1302.5)	5.76 ± 1.48	5.39 ± 1.43	0.107 (800.5)	6.04 ± 1.38	5.96 ± 1.33	0.470 (3116.5)
Results of hand hygiene observations helping to improve hand hygiene^g^	6.13 ± 0.91	6.29 ± 0.74	0.476 (1514.0)	5.96 ± 1.04	5.82 ± 0.87	0.309 (872.5)	5.84 ± 1.22	5.75 ± 1.30	0.697 (3168.5)
Impact of being observed and paying more attention to hand hygiene^g^	5.80 ± 1.43	6.09 ± 1.30	0.214 (1350.0)	5.54 ± 1.52	5.72 ± 0.98	0.891 (973.0)	5.01 ± 1.47	5.14 ± 1.80	0.287 (2930.5)
Importance of educational activities to improve one’s hand hygiene practices^h^	6.20 ± 1.09	6.27 ± 1.03	0.780 (1610.0)	5.70 ± 1.42	6.12 ± 0.73	0.377 (847.0)	5.29 ± 1.26	5.36 ± 1.37	0.505 (2934.5)
Perceived support from the management in improving hand hygiene^g^	6.41 ± 0.88	6.67 ± 0.69	0.045 (1352.5)	5.52 ± 1.35	5.84 ± 1.06	0.326 (895.0)	5.23 ± 1.48	5.09 ± 1.65	0.791 (3162.5)
Awareness of one’s role in preventing healthcare-associated infections by improving hand hygiene^g^	6.26 ± 1.15	6.54 ± 0.80	0.217 (1445.0)	6.17 ± 1.25	6.41 ± 0.76	0.733 (974.0)	5.95 ± 1.27	5.76 ± 1.55	0.683 (3242.5)
Perceived risk of patients developing a healthcare-associated infection^i^	28.03 ± 20.21	39.83 ± 26.10	0.016 (1105.0)	34.33 ± 21.67	37.62 ± 21.96	0.505 (951.5)	40.81 ± 23.79	39.74 ± 25.16	0.721 (2753.5)
How often healthcare workers comply with hand hygiene protocols^i^	76.35 ± 12.94	70.82 ± 26.60	0.716 (1264.5)	74.87 ± 14.14	77.60 ± 14.95	0.360 (858.0)	85.56 ± 11.34	80.08 ± 16.81	0.023 (2510.5)
Self-reported adherence to hand hygiene in required situations^i^	91.73 ± 9.34	89.36 ± 16.68	0.851 (1295.0)	92.27 ± 7.78	90.89 ± 10.13	0.780 (956.5)	92.89 ± 6.40	90.91 ± 10.54	0.568 (3072.5)

Note. Values in the 2020 and 2023 columns are presented as mean ± ± standard deviation. Comparisons between the 2020 and 2023 samples were performed separately within each participant group using the Mann–Whitney U test. For each comparison, the p value is reported together with the corresponding Mann–Whitney U value in parentheses. Response ranges:

a 1 = very low, 4 = very high

b 1 = low priority, 4 = very high priority

c 1 = not effective, 7 = very effective

d 1 = no importance, 7 = very high importance

e 1 = no effort, 7 = a big effort

f 1 = not at all, 7 = very well

g 1 = not at all, 7 = very much

h 1 = not at all, 7 = very important

i 0–100%.

Ratings for the survey item concerning the perceived importance of hand hygiene on a ward among all patient safety issues were lower across all groups in 2023 compared with 2020 (Table 3). For first-semester nursing students, the mean rating was lower in 2023 than in 2020 (3.30 vs 3.77, p < 0.001). The same pattern was observed among final-semester nursing students (2.89 vs 3.61, p < 0.001) and registered nurses (3.19 vs 3.39, p = 0.041). Additionally, perceived patient expectations regarding healthcare workers' hand hygiene were also lower in 2023 than in 2020 among first-semester nursing students (4.96 vs 5.55, p = 0.050) and final-semester nursing students (3.48 vs 5.04, p < 0.001).

Conversely, among first-semester nursing students, perceptions of the nursing programme’s emphasis on optimal hand hygiene were higher in 2023 than in 2020 (6.52 vs 6.13, p = 0.030). Ratings of perceived support from the nursing programme for hand hygiene improvement were also higher in 2023 (6.67 vs 6.41, p = 0.045), as were ratings of perceived skin tolerance of alcohol-based hand rubs (5.96 vs 5.35, p = 0.042).

Among first-semester nursing students, perceived risk of patients developing a healthcare-associated infection was higher in 2023 than in 2020 (39.83 vs 28.03, p = 0.016). Among registered nurses, perceived hand hygiene adherence among healthcare workers was lower in 2023 than in 2020 (80.08 vs 85.56, p = 0.023).

For other items, including perceived leadership support, availability of alcohol-based handrub, visibility of hand hygiene instructions, and the influence of educational activities, no statistically significant differences were observed between the 2020 and 2023 samples within any participant group.

## 4 Discussion

This study uniquely examined differences in hand hygiene knowledge and perceptions among nursing students and registered nurses between the peak of the COVID-19 pandemic (2020) and the post-pandemic period (2023). As the samples were not followed longitudinally, the findings should be interpreted as group-level differences between the two time points rather than individual-level changes over time. By comparing these time points, the study provides insights into the sustainability of pandemic-driven improvements in infection prevention and control practices. The main finding was significantly lower ratings for the survey item concerning the importance of hand hygiene as a patient safety measure in 2023 compared with 2020 across all groups. This finding should primarily be understood in relation to the heightened focus on infection prevention and control during the pandemic, when perceptions of hand hygiene were more positive across all groups (Blomgren et al., 2025), rather than as evidence of unsafe perceptions of hand hygiene in the post-pandemic period. The lower perception ratings in 2023 may therefore reflect a normalisation of risk perception after the peak of the pandemic. They may also suggest that, without sustained contextual pressure or institutional emphasis, the perceived importance of hand hygiene becomes less prominent, particularly in the absence of reinforcing structures. In relation to the theoretical framework adopted for this study (Ajzen, 1991; Hadziabdic et al., 2020), these findings may be interpreted as differences in perceptions of the value of hand hygiene, perceived expectations, and perceived support for performing hand hygiene. These findings support the perception-related aspect of our hypothesis that hand hygiene perceptions would differ between the pandemic and post-pandemic periods, particularly regarding perceived importance for patient safety, patient expectations and perceived support for performing hand hygiene.

### 4.1 Differences in hand hygiene knowledge and perceptions

Perceptions measured by the WHO survey (WHO, 2009), such as perceived importance, risk awareness, and institutional support, can be understood in line within our theoretical framework (Ajzen, 1991; Hadziabdic et al., 2020) as reflections of the underlying beliefs that guide attitudes and behaviour. The reduction in perceived hand hygiene importance among first- and final-semester nursing students and RNs suggests a potential shift in priorities or the effects of pandemic fatigue. Similar results have been reported in other studies, where prolonged exposure to high-intensity infection prevention and control measures has sometimes led to decreased adherence over time (Filip et al., 2022). While the temporary emphasis on infection prevention and control during the pandemic may have initially heightened awareness and adherence, the subsequent decline in attention to hand hygiene highlights the need for sustained educational reinforcement and organisational support to maintain infection prevention and control priorities as pandemic pressures subside (Shortage of personal protective equipment endangering health workers worldwide [Internet], 2024; Sarker et al., 2023; Copenhagen, 2024).

Lower perception scores do not necessarily mean that hand hygiene adherence was reduced, as this study measured knowledge and self-reported perceptions rather than observed practice. A meta-analysis of studies conducted during the early phase of the COVID-19 pandemic, from January 2020 to October 2021, showed that observed hand hygiene compliance varied between professional groups, with compliance estimated at 80% among nurses, 76% among doctors, and 70% among other healthcare workers (Wang et al., 2022). However, because the present study did not include observational data, it cannot determine whether the lower perception scores observed in 2023 corresponded to lower clinical hand hygiene adherence. The findings may instead reflect that hand hygiene became less prominent in everyday risk perception after the peak of the pandemic. Nevertheless, the affected items from the WHO Perception Survey (WHO, 2009), including perceived importance for patient safety, patient expectations, and perceived adherence among healthcare workers, have clinical relevance. Lower ratings of hand hygiene importance may indicate that it is perceived as less central in daily safety work. Reduced perceived patient expectations may limit patients’ role as active partners in infection prevention, while lower perceived adherence among healthcare workers may indicate a continued need for feedback, reminders, and visible role modelling in clinical settings.

Taken together, the findings can be mapped onto the three components of the Theory of Planned Behaviour (Ajzen, 1991). First, the lower rating of hand hygiene as a patient safety measure reflects the attitude component, as it concerns beliefs about the value and importance of the behaviour. Second, reduced perceived patient expectations and perceived adherence among healthcare workers relate to subjective norms, as they concern perceived expectations and visible hand hygiene practices among others. Third, increased perceptions of educational support and better tolerance of alcohol-based hand rubs among first-semester students relate to perceived behavioural control, as they may influence how easy and supported hand hygiene is perceived to be. Thus, the results suggest that post-pandemic differences among nursing students and registered nurses were not limited to knowledge but also involved beliefs linked to attitudes, normative expectations and perceived control.

Nursing students, particularly those in their first semester, reported increased recognition of the nursing programme's support for hand hygiene practices in 2023 compared with 2020, as well as higher ratings of perceived skin tolerance of alcohol-based hand rubs. This aligns with findings that positive reinforcement and institutional support can foster favourable attitudes towards hand hygiene (World Health Organization, 2024; Luangasanatip et al., 2015). These findings suggest that educational institutions may play a role in shaping how hand hygiene is perceived by nursing students. In line with the theoretical framework applied in this study (Ajzen, 1991, Hadziabdic et al., 2020), nursing education not only imparts knowledge but also establishes interpretive frameworks through which students understand infection prevention and control priorities. Both the content of the curriculum and the informal learning that occurs during clinical placements may contribute to how students perceive when and why hand hygiene is important. The WHO “Multimodal Hand Hygiene Improvement Strategy”, reaffirmed in the 2024 Global Report on Infection Prevention and Control (World Health Organization, 2024), highlights supportive leadership, education, reminders, and institutional safety culture as key elements for sustaining long-term hand hygiene adherence. These principles are also supported by findings from a systematic review that applied a behavioural perspective to hand hygiene improvement strategies (Luangasanatip et al., 2015). While institutional influence appears to support favourable perceptions of hand hygiene, the lower ratings of hand hygiene importance in 2023 suggest that such gains may not be maintained without ongoing reinforcement and structured feedback (Blomgren et al., 2021; Sax et al., 2007; Braithwaite et al., 2017).

Among registered nurses, the lower ratings of perceived hand hygiene adherence among healthcare workers in 2023 align with previous studies showing that feedback and visible adherence by peers and supervisors may support hand hygiene practice (World Health Organization, 2024). As previous research has demonstrated, nursing students often look to registered nurses to model infection prevention behaviours, making the visibility of adherence among registered nurses relevant to how students perceive and learn infection prevention practices (Zimmerman et al., 2020; Barrett and Randle, 2008; Labrague et al., 2018). This finding underscores the professional responsibility highlighted in the International Council of Nurses’ Code of Ethics (ICN - International Council of Nurses [Internet], 2023), which emphasises nurses’ duty to safeguard patients, act as role models for safe care, and collaborate with colleagues to uphold infection prevention and control standards.

Another notable finding was the increased perceived risk of healthcare-associated infections among first-semester students. This likely reflects the residual impact of intensified infection prevention and control messaging during the pandemic. The WHO's Perceptions Survey for Health-Care Workers (WHO, 2009) and other tools have consistently shown that healthcare workers' awareness of healthcare-associated infections risks can promote adherence to hand hygiene protocols, as heightened risk perception is often correlated with proactive infection prevention and control behaviours. The WHO survey emphasises that risk awareness, when combined with regular feedback and a supportive environment, strengthens healthcare workers' commitment to hand hygiene as an essential preventive measure (World Health Organization, 2024; Kusain and J, 2015).

### 4.2 Post-pandemic challenges

In the Swedish healthcare context, staffing shortages and high turnover following the pandemic have reduced workplace continuity, with potential consequences for adherence to infection prevention and control practices. Moreover, increased reliance on temporary and agency nurses has introduced additional challenges, as these nurses may not be fully integrated into local infection prevention and control protocols or hospital routines. This lack of familiarity can lead to inconsistencies in hand hygiene compliance and create an additional workload for permanent staff, who must also supervise and support temporary personnel (Rosenbäck et al., 2022).

Many experienced nurses left the profession due to burnout and pandemic-related stress, further disrupting mentorship structures and peer reinforcement of hand hygiene behaviours (Bäckström et al., 2024; ICN Policy Brief_Nurse Shortage and Retention_0.pdf [Internet], 2025). The absence of experienced senior nurses may have weakened the transmission of infection prevention and control practices, particularly among newly trained staff. This is consistent with observations that many healthcare facilities still lack ongoing training and strong leadership support for infection prevention and control implementation (World Health Organization, 2024).

From a theoretical perspective, the framework adopted in this study (Ajzen, 1991; Hadziabdic et al., 2020) suggests that hand hygiene perceptions may be influenced by institutional and social influences, such as curricula, clinical mentors and peer expectations. These contextual factors may contribute to how hand hygiene is perceived and understood in everyday care. When institutional continuity is disrupted, for instance, by staff turnover or the absence of senior role models, the social reinforcement of hand hygiene-related beliefs may be weakened, thereby undermining both attitudes and perceived behavioural control. Within this framework, access to experienced colleagues strengthens perceived behavioural control by enhancing competence and confidence in hand hygiene performance.

These workforce-related factors may provide one possible context for understanding the lower ratings of perceived hand hygiene adherence among registered nurses in 2023. The visibility of role models may be important for maintaining shared expectations regarding infection prevention and control. However, when senior nurses are absent or replaced by temporary staff, this continuity may be disrupted. Within the theoretical framework adopted in this study (Ajzen, 1991; Hadziabdic et al., 2020), such disruptions may be relevant to subjective norms, understood here as perceived social and professional expectations regarding hand hygiene behaviour. Previous research suggests that nursing students and junior nurses often adopt behaviours from their senior colleagues; consequently, staffing instability may be relevant to consider when interpreting hand hygiene perceptions in clinical settings (Zimmerman et al., 2020; Barrett and Randle, 2008; Braithwaite et al., 2017).

### 4.3 Clinical and educational implications

The findings of this study suggest several areas that may warrant attention in nursing education and clinical practice. In nursing education, the item-level knowledge differences observed among first-semester nursing students in 2023 suggest that selected aspects of hand hygiene knowledge, particularly transmission routes and preventive actions, may require continued reinforcement. This could include explicit discussion of the items that differed between 2020 and 2023, including the main route of cross-transmission, routine use of alcohol-based hand disinfectants, and the importance of avoiding jewellery and artificial fingernails during patient care. In clinical settings, registered nurses’ lower ratings of healthcare workers’ hand hygiene adherence may indicate a need to monitor how hand hygiene practices are perceived and discussed in everyday clinical work.

Continuing education may be particularly important for aligning nursing education and clinical practice in relation to hand hygiene. In Sweden, nursing education and continuing education for healthcare workers are organised by different institutions, which may create variation in terminology, emphasis and expectations regarding hand hygiene. Closer collaboration between nursing programmes and clinical units could therefore support a shared understanding of hand hygiene principles and expectations. This may be particularly relevant considering the lower ratings in 2023 for perceived hand hygiene importance, perceived patient expectations and perceived adherence among healthcare workers.

Nursing programmes and clinical units could use joint seminars, structured discussions or regular feedback sessions to align educational content with clinical routines and reinforce hand hygiene as part of patient safety. Outcomes could be evaluated over time by monitoring hand hygiene knowledge and perceptions and, where possible, observed adherence through repeated surveys, audits and/or direct observations.

A further clinical implication concerns mobile device use in clinical settings, which is not captured by the WHO instruments. Although mobile phone use was not assessed in the present study, previous research has shown that mobile phones are frequently used during clinical work and may act as reservoirs for microorganisms (Galazzi et al., 2019; Simmonds et al., 2020). These findings are relevant to hand hygiene because repeated handling of mobile devices during clinical work may contribute to hand recontamination and affect infection prevention and control routines. Future hand hygiene research and educational initiatives should therefore consider mobile device handling, cleaning routines and hand hygiene in connection with mobile phone use during clinical work.

### 4.4 Limitations

One limitation of this study is its comparative cross-sectional survey design with two time points and independent samples, which does not permit inferences about individual-level change over time (Polit and Beck, 2021). Consequently, the observed differences between 2020 and 2023 should be interpreted as population-level shifts rather than longitudinal developments within the same individuals. However, the comparative cross-sectional design adopted in this study offers an efficient way to examine differences between groups at distinct time points (Polit and Beck, 2021), contributing important knowledge from an unforeseen emergency such as the COVID-19 pandemic that would otherwise not have been captured.

Another limitation of this study is the overall response rate of 39.6%, with lower participation among registered nurses (30% in 2020 and 36% in 2023), which introduces the possibility of selection bias. Non-responders may differ systematically from responders, which could affect the generalisability of the results (Petrie and Sabin, 2020; Polit and Beck, 2021). For instance, if eligible participants with lower interest in hand hygiene or less confidence in their knowledge were less likely to respond, the sample may disproportionately represent individuals with more positive perceptions or stronger knowledge. This could result in an overestimation of knowledge and perceptions in the findings.

This selection bias may also have affected the observed differences between 2020 and 2023. For example, if those who responded in 2023 were less interested in hand hygiene than those who responded in 2020, the lower perceived importance and perceived adherence may partly reflect differences in the respondent groups rather than true differences between the two time points. In addition, the 2023 data collection coincided with the period when summer workers usually receive their workplace introduction, which may have influenced the clinical context. Since no individual-level information was available for non-responders, it was not possible to formally compare responders with the eligible population to assess representativeness.

Moreover, the lower response rate among first-semester students in 2023 (35%) compared with 2020 (56%) may reflect contextual influences on participation. During the pandemic, hand hygiene was a highly pressing and urgent issue, which may have motivated higher participation. In contrast, the 2023 survey was conducted just before the summer holidays, when students may have been less available or less motivated to engage with a topic perceived as less urgent in the post-pandemic period (Polit and Beck, 2021).

The reliance on self-reported data rather than direct observations also represents a limitation. While surveys provide valuable insights into perceptions and knowledge, they may not fully capture actual hand hygiene behaviour in practice. Self-reported compliance often diverges from observed practice, underscoring the need to interpret these findings with caution (van Dijk et al., 2023). Future studies should therefore combine measures of knowledge and perceptions with direct observations or audit data to examine how these factors relate to actual hand hygiene adherence and whether changes in perceptions are reflected in clinical behaviour over time.

Despite these limitations, the study has notable methodological strengths. By including both first- and final-semester nursing students alongside clinically active registered nurses, the study captures a broad range of professional experience and educational exposure, providing insights into how hand hygiene perceptions may have shifted in response to changing infection prevention and control conditions (Polit and Beck, 2021). Furthermore, the use of the WHO’s validated hand hygiene instruments, translated using the WHO’s recommended forward–backward translation method (World Health Organization) and pilot-tested in the Swedish context, strengthens the validity, reliability and contextual relevance of the findings (Polit and Beck, 2021).

## 5 Conclusion

This study partly supports the hypothesis that nursing students’ and registered nurses’ knowledge and perceptions of hand hygiene differed between the peak of the COVID-19 pandemic and the post-pandemic period, particularly regarding perceptions. However, because of the independent-sample design, these differences should be interpreted at the group level and not as individual-level change over time. While first-semester nursing students reported greater recognition of hand hygiene support within their educational programmes in 2023, the lower perceived importance of hand hygiene across groups may reflect a normalisation of risk perception after the pandemic, rather than direct evidence of reduced hand hygiene adherence. Total knowledge scores did not differ significantly between 2020 and 2023 within any group, although item-level knowledge differences were observed among first-semester nursing students.

The findings indicate that hand hygiene perceptions may be less prominent in the post-pandemic period and identify specific areas for educational and clinical follow-up. These include selected knowledge items among first-semester nursing students, perceived hand hygiene importance, perceived patient expectations and perceived adherence to recommended hand hygiene practices among healthcare workers. Future research should combine survey-based measures with direct observations or audit data to clarify how knowledge and perceptions relate to clinical hand hygiene practice.

## Funding statement

This research received no specific grant from any funding agency in the public, commercial, or not-for-profit sectors.

## CRediT authorship contribution statement

**Per-Ola Blomgren:** Writing – review & editing, Writing – original draft, Visualization, Validation, Methodology, Investigation, Formal analysis, Data curation, Conceptualization. **Lisa Hultin:** Writing – review & editing, Visualization, Validation, Supervision, Methodology, Investigation, Formal analysis, Data curation, Conceptualization. **Johan Westerbergh:** Writing – review & editing, Visualization, Formal analysis, Data curation. **Katarina Hjelm:** Writing – review & editing, Visualization, Validation, Supervision, Resources, Project administration, Methodology, Investigation, Funding acquisition, Formal analysis, Data curation, Conceptualization.

## Declaration of competing interest

All authors declare that they have no conflicts of interest.

## Appendix Supplementary materials

Image, application 1
