# Supplementary material for: Differences in hand hygiene knowledge and perceptions among nursing students and registered nurses during and after the COVID-19 pandemic: A comparative cross-sectional survey
Source: Int J Nurs Stud Adv. 2026 Jul 20;11:100639. doi: 10.1016/j.ijnsa.2026.100639 (PMC13418222; doi:10.1016/j.ijnsa.2026.100639)
Supplement: Supplementary file 1 [file mmc1.docx]

| **Supplementary Table 1**. Overview of infection prevention and control content and focus in the nursing programme (years 1–3) and registered nurses’ workplace. | | | | |
| --- | --- | --- | --- | --- |
|  | 1^st^ year (semesters 1–2) | 2^nd^ year (semesters 3–4) | 3^rd^ year (semesters 5–6) | Registered nurses’ workplace |
| infection prevention and control training content | Routes of transmission, hand hygiene, dress code, cleaning/disinfection of surfaces and medical equipment, sterilisation, waste management. | Prevention of transmission in healthcare environments, healthcare-associated infections, repetition of transmission routes (including airborne infection), repetition of hand hygiene and dress code, cleaning, disinfection, and sterilisation. | Covers all infection prevention and control aspects from previous years with a focus on work management and qualitative. | The WHO’s Multimodal Hand Hygiene Improvement Strategy, hand hygiene infrastructure, training tools, evaluation/feedback, reminders, and patient safety initiatives. |
| Focus | Supports students in initial clinical placements by introducing core infection prevention and control principles, hands-on patient care, and healthcare-associated infections prevention. | Reinforces key infection prevention and control principles and expands on airborne transmission. | Emphasises infection prevention and control in work organisation and quality improvement. | Registered nurses must complete an annual interactive infection prevention and control training programme via the intranet. |
